# Segment Anything Model (SAM) and Medical SAM (MedSAM) for Lumbar Spine MRI

**DOI:** 10.3390/s25123596

**Published:** 2025-06-07

**Authors:** Christian Chang, Hudson Law, Connor Poon, Sydney Yen, Kaustubh Lall, Armin Jamshidi, Vadim Malis, Dosik Hwang, Won C. Bae

**Affiliations:** 1 Punahou School, Honolulu, HI 96822, USA; 2 The Cambridge School, San Diego, CA 92129, USA; 3 Polytechnic School, Pasadena, CA 91106, USA; 4 Valencia High School, Santa Clarita, CA 92870, USA; 5 ResMed Inc., San Diego, CA 92123, USA; kaustubhlall@gmail.com; 6 Radicle Science, Encinitas, CA 92024, USA; 7 Department of Radiology, University of California-San Diego, San Diego, CA 921093, USA; 8 Department of Electrical and Electronic Engineering, Yonsei University, Seoul 03722, Republic of Korea; dosik.hwang@yonsei.ac.kr; 9 Center for Healthcare Robotics, Korea Institute of Science and Technology, Seoul 02792, Republic of Korea; 10 Department of Radiology, Research Institute of Radiological Science and Center for Clinical Imaging Data Science, Yonsei University College of Medicine, Seoul 03722, Republic of Korea; 11 Department of Oral and Maxillofacial Radiology, Yonsei University College of Dentistry, Seoul 03722, Republic of Korea; 12 Department of Radiology, VA San Diego Healthcare System, San Diego, CA 92161, USA

**Keywords:** intervertebral disc, vertebral body, artificial intelligence, deep learning, vision transformer, image segmentation, promptable, zero shot

## Abstract

Lumbar spine Magnetic Resonance Imaging (MRI) is commonly used for intervertebral disc (IVD) and vertebral body (VB) evaluation during low back pain. Segmentation of these tissues can provide useful quantitative information such as shape and volume. The objective of the study was to determine the performances of Segment Anything Model (SAM) and medical SAM (MedSAM), two “zero-shot” deep learning models, in segmenting lumbar IVD and VB from MRI images and compare against the nnU-Net model. This cadaveric study used 82 donor spines. Manual segmentation was performed to serve as ground truth. Two readers processed the spine MRI using SAM and MedSAM by placing points or drawing bounding boxes around regions of interest (ROI). The outputs were compared against ground truths to determine Dice score, sensitivity, and specificity. Qualitatively, results varied but overall, MedSAM produced more consistent results than SAM, but neither matched the performance of nnU-Net. Mean Dice scores for MedSAM were 0.79 for IVDs and 0.88 for VBs, and significantly higher (each *p* < 0.001) than those for SAM (0.64 for IVDs, 0.83 for VBs). Both were lower compared to nnU-Net (0.99 for IVD and VB). Sensitivity values also favored MedSAM. These results demonstrated the feasibility of “zero-shot” DL models to segment lumbar spine MRI. While performance falls short of recent models, these zero-shot models offer key advantages in not needing training data and faster adaptation to other anatomies and tasks. Validation of a generalizable segmentation model for lumbar spine MRI can lead to more precise diagnostics, follow-up, and enhanced back pain research, with potential cost savings from automated analyses while supporting the broader use of AI and machine learning in healthcare.

## 1. Introduction

Low back pain (LBP) affects millions worldwide and has severe economic consequences due to healthcare costs, lost productivity, and disability [1]. LBP is a multi-factorial disease, where an alteration in any of the tissues near the lumbar spine may cause the pain. Among these, degeneration of intervertebral discs (IVD) [2] and vertebral bodies (VB) [3] have been implicated in LBP, and clinical imaging such as magnetic resonance imaging include the evaluation of these tissues as the routine protocol [4,5,6,7,8,9,10,11,12]. MRI protocols including sagittal spin echo T2-weighted [13] sequences are often used for evaluation of both the IVD degeneration [12] as well as alteration in VB bone and marrow.

While MRI images are typically evaluated as a whole by a radiologist familiar with the anatomy, for advanced quantitative techniques such as T2 mapping [14,15] where MR properties of the tissues are being measured, regions of interest (ROI) are drawn around each tissue (i.e., segmentation) to determine the tissue-specific T2 value. Additionally, measurement of tissue volume [16] or shape [17,18] also require segmentation.

Conventionally, automated segmentation of these tissues has been challenging. As apparent on typical spin echo MRI images of a lumbar spine (Figure 1), the boundaries of the IVD and VB can be irregular, and the signal intensity of the contiguous tissue is often inhomogeneous. The classical methods included utilizing histogram-based voxel intensity to segment the image via thresholding [19,20]. Another conventional method is region-growing, where a seed point is placed and grown based on the similarity of the voxel intensity will fill an ROI [21,22,23].

With the advent of deep learning (DL) models such as full convolutional network (FCN) [24], U-Net [25], and Google DeepLab [26] have shown good results in medical image segmentation, reaching Dice scores of 85% or higher when segmenting the IVD [27,28]. In a study by Wang et al., [29] a 3D DeepLab was used to segment multiple lumbar structures, including vertebrae, intervertebral discs, nerve roots, and muscles, achieving high Dice scores > 0.85. Another study [30] developed a deep neural network for automatic segmentation of lumbar muscles (e.g., psoas major, multifidus) in axial MRI scans, validated on external datasets. Similarly, [31] demonstrated the feasibility of convolutional neural networks for fully automated segmentation of vertebral bodies, discs, and paraspinous muscles, with results comparable to human-demarcated masks with disc scores ≥ 0.77. Building upon basic U-Net [32,33], boundary specific U-Net [27] that uses a modified loss function for the boundary of the segmentation rather than the area and MultiResUNet [34,35] that uses multiple convolutional layers with different kernel sizes to capture features with varying spatial resolutions have been used for improved intervertebral disc segmentation. Sharp U-Net [36] is another enhanced variant of U-Net that utilizes a depthwise convolutional sharpening filter on the encoder’s feature maps before merging them with the decoder’s features. This achieved matching or better results compared to a plain U-Net with the same number of parameters when segmenting images from different modalities. Using U-Net’s basic architecture but focusing on refined automatic configuration and training rather than introducing new architecture, nnU-Net [37] (“no new net”) was found to perform well for segmenting a variety of organs when compared against existing models. However, nnU-Net’s performance on musculoskeletal images have not yet been reported.

These models, while varied in architecture, all require supervised learning with a training data that is highly specific in terms of image appearance (e.g., lumbar spine MRI with a particular weighting) and the task (e.g., segmentation of only IVD), rendering them ungeneralizable to other images and tasks. Recently, promptable foundation segmentation DL models have been introduced, such as Segment Anything Model (SAM) [38] developed by the company META, and a fine-tuned adaptation of SAM called medical SAM (MedSAM) [39] that was trained on additional medical image segmentation pairs. At a high-level description, these models build on Transformer vision models [40,41] with an image encoder, prompt encoder, and mask decoder. These pre-trained “zero-shot” models are meant to be used to perform segmentation across multiple types of images and objects, only using a minimal user input prompt such as placement of point(s) or a bounding box around the object of interest.

For broader adaptation and usage of different DL models, validation and understanding of model behavior under different conditions is needed. SAM and MedSAM have not been tested in-depth for lumbar spine MRI segmentation of IVD and VB. MRI images acquired with different scan parameters have also not been used. The purpose of this study was to (1) compare the performance metrics of SAM, MedSAM, and nnU-Net for the segmentation of intervertebral discs (IVD) and vertebral bodies (VB) in lumbar spine MRI, (2) determine how image contrast affects segmentation metrics, and (3) determine inter-reader agreement in segmentation metrics for SAM and MedSAM.

## 2. Materials and Methods

**IRB Statement:** This cadaveric study was exempted from an Institutional Review Board approval. Informed written consents were not required.

**Study Design:** This is a prospective cross-sectional study to compare the performance metrics of segmentation of IVD and VB from lumbar spine MRI, when using different DL models.

**Spine Specimens:** MRI images from 82 cadaveric lumbar spines including at least L1 to L5 vertebrae were acquired. The specimens were from 64 males and 18 females, ages between 24 and 75 years old, with a mean and standard deviation of 57.5 and 10.4 years old, respectively.

**MRI:** MRI data from a recently published work [42] was used. Imaging was performed on a 3-T system (GE Signa HDx) with an 8-channel transmit/receive knee coil. Imaging protocol was sagittal fast spin echo (FSE) with constant repetition time (TR) and variable echo time (TE) to acquire eight images suitable for T2 mapping (Figure 1). This opportunistic data provided a great opportunity to determine the effect of varying TE on the performance of deep learning models. The following scan parameters were used: sagittal imaging plane through the center of the spine, TR = 2000 milliseconds (ms), eight TEs = 8, 16, 24, 32, 40, 48, 56, and 64 ms, field of view (FOV) = 180 to 220 mm, image matrix = 256 × 256, and slice thickness = 3 millimeters (mm). This MRI sequence is similar to a routine sagittal T2 sequence used in clinical settings [43]. Note the change in image appearance with TE: at the first TE of 8 ms, the signal intensity for all tissues is the highest, while the IVD shows the most homogenous signal intensity; at the highest TE of 64 ms, IVD shows distinctly high signal intensity for the nucleus pulposus, NP, and a low signal intensity for the annulus fibrosus, AF.

**Prompts for SAM and MedSAM:** To perform segmentation with SAM, two prompting methods were used. One was a point-input within each target region and the background was needed. Two readers (high school students) with no previous experience were trained by the senior investigator (W.C.B. with over 10 years of experience) in MRI sectional anatomy of the lumbar spine and tasked to placing points within each IVD and VB on the first TE image (proton density-weighted), as well as the background (Figure 2A). The same readers performed the second task, which was to draw multiple boxes encompassing the IVD and VB (Figure 2B). MedSAM also accepts the box input (but not the point-input), so the same box inputs were used. These input prompts were created using ImageJ [44] (v2.1.0), and the coordinates of the points and boxes were saved for further processing. Although the readers were inexperienced, given the simple nature of placing points or bounding boxes, the readers had no trouble completing the task correctly when checked by the senior investigator.

**Ground Truth Segmentation:** All images were manually segmented using ImageJ [44] (v2.1.0) by a senior investigator (W.C.B.) with over 10 years of experience in spine MRI research. Regions of interest (ROI) representing the entire IVD and VB were drawn on the first TE image. The boundaries of the IVD, including both the NP and AF, were the superior and inferior endplates, and anterior and posterior longitudinal ligaments (Figure 2C). The boundaries of the VB were the longitudinal ligaments and the IVDs (Figure 2D).

**Figure 3:**
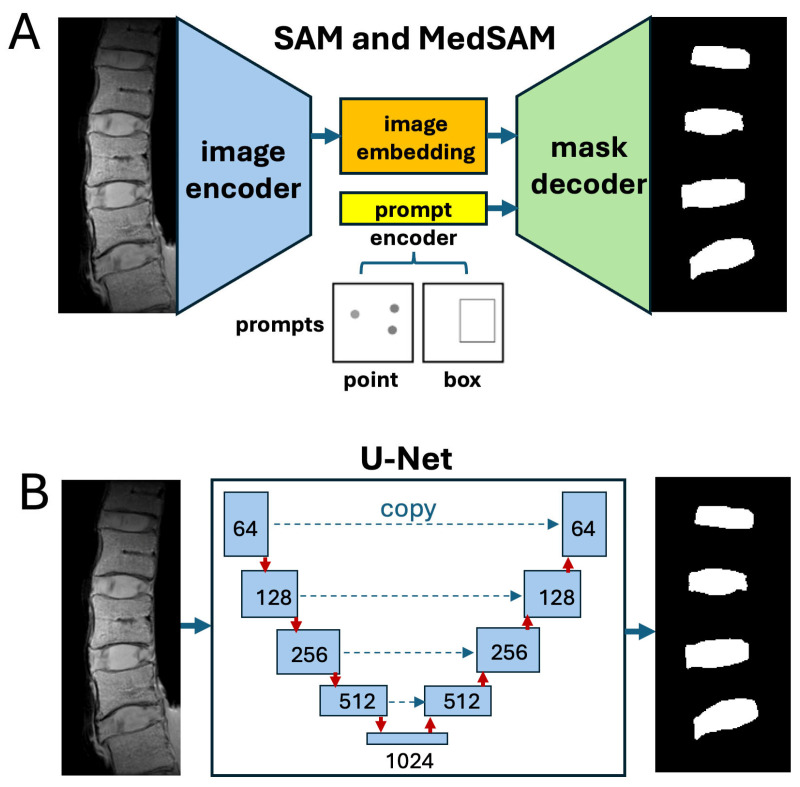
Deep learning models used in this study. (**A**) SAM and MedSAM have components of image encoder, prompt encoder, and a mask decoder that creates the segmented mask. (**B**) U-Net, basis for nnU-Net, has layers of image encoders and decoders with skip connection to retain high spatial details.


**Figure 4:**
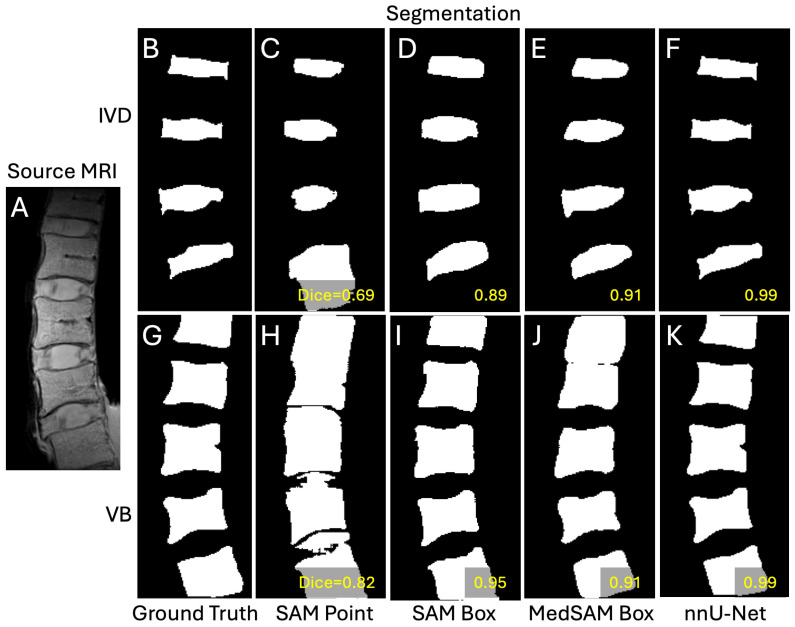
A representative case showing the input image (**A**), ground truths (**B**,**G**), segmentations from DL models of SAM with point prompts (**C**,**H**), SAM with box prompts (**D**,**I**), MedSAM with box prompts (**E**,**J**), and nnU-Net (**F**,**K**). Top row (**B**–**F**) are intervertebral discs and the bottom row (**G**–**K**) are vertebral bodies.

**Deep Learning Models:** For SAM and MedSAM (Figure 3A), we utilized pre-trained built-in models available on Matlab with Image Processing Toolbox (R2024b). The models and MRI images were loaded onto the program, and segmentation for each IVD and VB were output based on the coordinates of the points (for SAM) or the boxes (for MedSAM). The outputs were segmented images of the IVD (Figure 4C–E) and VB (Figure 4H–J). Processing (inferencing) time for SAM and MedSAM were ~7.5 and ~10 s per image, respectively, on a MacBook Pro with M1 Pro processor and 16 GB of RAM (Apple Inc., Cupertino, CA, USA). The following is a pseudo-code for performing SAM or MedSAM segmentation, using either a point or a box input.

I = imread('input image'); imageSize = size(imtemp); model = SegmentAnythingModel; % model = medicalSegmentAnythingModel; % for MedSAM embeddings = extractEmbeddings(model,I); points = 'coordinates for point prompt for IVD or VB' backgroundPoints = 'coordinate for background point' box =  'coordinates for a bounding box prompt for IVD or VB' % using point input segmentation = segmentObjectsFromEmbeddings(model,embeddings,imageSize, ...                 ForegroundPoints=points,BackgroundPoints=backgroundPoints); % using box input segmentation = segmentObjectsFromEmbeddings(model,embeddings,...                         imageSize,BoundingBox=box);


For the U-Net (Figure 3B), we utilized the state-of-the-art iteration of U-Net called nnU-Net [37]. A U-Net was chosen based on our past experience with it, robust performance for many applications, and its wide usage since inception. Briefly, the nnU-Net (the newest v2 dated 18 April 2024, available at https://github.com/MIC-DKFZ/nnUNet, accessed on 10 April 2025) automatically adapts to a given data, by analyzing the training cases and automatically configuring a matching U-Net pipeline. We used a 70/15/15 split for training/validation/testing, equal to 460/98/98 images. For preprocessing, the images were converted to a compressed nitfi format, and the labels were rescaled to have intensity values of 0, 1, and 2 for the background, discs, and vertebral bodies, respectively. Training was performed on a Windows 10 PC with Intel core i9-9900K CPU, 32 GB RAM, and a NVIDIA RTX2080 GPU with 8 GB VRAM and took ~2 days. The automatically configured model was as follows: 2D U-Net with 6 stages with progressively increasing feature channels (32, 64, 128, 256, 512, 512), each stage using two convolutional layers with 3 × 3 kernels and strides that downsample spatial dimensions at each level after the first. The decoder mirrors this configuration with symmetric upsampling. All convolution operations use 2D convolutions with instance normalization, Leaky ReLU activation functions, and no dropout. The model was trained with a batch size of 25 using 224 × 256 patches extracted from images normalized using z-score normalization. The architecture used batch Dice loss. For direct comparison against SAM and MedSAM, the entire dataset was inferenced to create segmented images of the IVD and VB, rather than inferencing just the test data. The inferencing, also performed on the PC, took ~1 s per image.

**Segmentation Metrics:** DL segmentations from SAM, MedSAM, and nnU-Net were compared against the ground truth segmentation using the following metrics. Dice score [37,38] provided a measure of image (i.e., segmentation mask) overlap, defined in Equation (1):

(1) $$Dice=\frac{2TP}{2TP+FP+FN}$$

where TP is the number of true positive voxels (i.e., value of 1 in both DL and ground truth segmentations), FP is the number of false positive voxels (value of 1 in DL, value of 0 in ground truth), and FN is the number of false negative voxels (value of 0 in DL, value of 1 in ground truth).

Sensitivity [39] was determined as in Equation (2):

(2) $$Sensitivity=\frac{TP}{TP+FN}$$

Specificity [39] was determined as in Equation (3):

(3) $$Specificity=\frac{TN}{TN+FP}$$

where TN is the number of true negative voxels (value of 0 in both DL and ground truth).

Lastly, robust Hausdorff distance (RHD) [45] between the DL segmentation and the ground truth was determined from the boundary points. Let A = {a_1_, …, a_m_} and B = {b_1_, …, b_n_} be the boundary point sets from the segmentations. The RHD between two sets of boundary points is a variation of the classical Hausdorff distance that reduces sensitivity to outliers. It uses the 95th percentile of minimum distances from each point in one set to the nearest point in the other set. Formally, let $d\left(a,B\right)=\underset{b\in B}{\min}\left|\right|a-b||$ be the distance from point a∈A to set *B*. Then the directed robust Hausdorff distance from *A* to *B* is defined as follows:

(4) $$RHD\left(A,B\right)=quantile_{95\%}\left(\left\{d\left(a,B\right)|a\in A\right\}\right)$$

**Statistics:** Segmentation metrics (Dice score, sensitivity, and specificity) of the four DL models (SAM with point input, SAM with box input, MedSAM with box input, and U-Net) to segment IVD and VB on the first echo image (TE = 8 ms; proton density-weighted) were compared using either Friedman statistic (for Dice, sensitivity, and specificity) or repeated measures analysis of variance (rmANOVA; for RHD) with post hoc comparisons. The effect of echo time of the source image on the Dice score was determined using Friedman statistic with post hoc comparisons, separately for each DL model and tissue (IVD and VB). The inter-reader agreement of Dice score was determined via intraclass correlation coefficient (ICC), for each DL model and tissue. For all tests, the significance level was set at 5%. Statistical analyses were performed using JASP software (version 0.18.3, jasp-stats.org).

## 3. Results

**Effect of DL Model:** Quantitatively, mean segmentation metrics varied by DL model (Table 1). The mean Dice scores for the IVD was the lowest for SAM with point prompts (0.64 ± 0.20, mean ± standard deviation), and higher for SAM with box prompts (0.83 ± 0.12), MedSAM (0.79 ± 0.11), and nnU-Net (0.99 ± 0.22). Dice scores for the VB were comparatively higher: for SAM (point), SAM (box), MedSAM, and nnU-Net, the values were 0.83 ± 0.07, 0.86 ± 0.05, 0.88 ± 0.04, and 0.99 ± 0.02, respectively. Post hoc analysis revealed significant (each *p* < 0.05) differences between every pairwise comparison. Similar trends were found with sensitivity values, while the specificity values were very high (~0.99) across all DL models. RHD values (where a higher value indicates worse match between boundary points) showed a similar trend as Dice scores: IVD RHD for SAM (point), SAM (box), MedSAM, and nnU-Net, were 7.4 ± 3.6, 4.1 ± 1.3, 4.6 ± 1.3, and 0.5 ± 0.7, respectively. The values were slightly higher for VB but with a similar trend across DL models. The differences in Dice, sensitivity, and RHD values were attributable to false negative inferences, i.e., over-segmentation.

**Observations:** Qualitatively, the segmentation results varied and were imperfect in many cases, often grossly over-segmenting the intended region (e.g., Figure 4C,H,J). However, there were many cases that nearly matched the ground truth segmentation (e.g., Figure 4D–F,I,K). A notable observation was that SAM and MedSAM segmentations tended to have smooth/rounded corners, while nnU-Net maintained sharp features better. Between the point and box prompts used for SAM, the point prompts tended to result in frequent over-segmentation (Figure 4C,H), while the box prompts appeared to mitigate this problem and result in better isolation of IVDs and VB regions. Between SAM and MedSAM (both box prompts), the segmentation appeared similar, both performing better than SAM using the point prompt. Here, nnU-Net performed the best, with the fewest instances of over-segmentation. Nonetheless, there were few exceptions where SAM with point prompts performed the better than SAM and MedSAM models (Figure 5).

**Effect of TE:** There was a significant effect of the TE on the Dice scores (Figure 6 and Table 2 showing the *p*-values), but the manifestation varied by the DL model. Figure 5A shows an example of IVD segmentation performed with SAM using point prompts at varying TEs. For SAM using point prompts (Figure 6B,C, yellow circle), the Dice score generally increased with TE, being the lowest at TE1 and TE2. This effect was more obvious for the IVD (Figure 6B, yellow circle; *p* < 0.001) than the VB (Figure 6C, yellow circle; *p* = 0.008). For SAM using box prompts, there was a slightly decreasing Dice score with TE for the IVD (Figure 6B, blue circles; *p* < 0.001), but a slightly increasing Dice score with TE for the VB (Figure 6C, blue circles; *p* = 0.01). For MedSAM, no change in Dice score with TE was found for the IVD (Figure 6B, pink circles; *p* = 0.27) but an increase Dice score with TE was found for the VB (Figure 6C, pink circles; *p* < 0.001). For nnU-Net, Dice score averaged above 0.98 across all TEs. While it varied with TE for the IVD (Figure 6B, green circles; *p* < 0.001) and VB (Figure 6C, green circles; *p* < 0.001) no notable trends were found.

**Inter-reader Agreement:** Figure 7 shows that intraclass correlation coefficients (ICC) of Dice scores of two readers, which were good-to-excellent (0.757 to 0.98) across different DL models and tissue. SAM with point prompts (Figure 7A,D) had inconsistent ICC (excellent for IVD at 0.926 and good for VB at 0.757) depending on the tissue. SAM with box prompts (Figure 7B,E) and MedSAM (Figure 7C,F) had consistently high ICC values > 0.8, and many data points along the identity line where the measurements agree perfectly.

## 4. Discussion

These results demonstrate the feasibility of “zero-shot” DL models to segment lumbar spine MRI and compared the performance against nnU-Net. For IVD, while SAM with point prompts performed significantly worse (mean Dice = 0.64), SAM with box prompts performed (Dice = 0.83) slightly better than MedSAM (Dice = 0.79). nnU-Net performed the best, with Dice score averaging over 0.98. For VB, Dice scores of all models improved to above 0.8, presumably due to a much larger segmented area compared to the IVD. SAM with point prompts still performed the worst, with an average Dice = 0.83, but not too far behind SAM with box prompts (Dice = 0.86), MedSAM (Dice = 0.88), and nnU-Net (Dice = 0.99). A lower performance of SAM with point prompts vs. SAM and MedSAM with box prompts appears attributable largely to the differences in the input; a bounding box input appears to hinder (but does not necessarily eliminate) over-segmentation beyond the boundary of the box. Between SAM vs. MedSAM with box prompts, the performances were similar; SAM performed slightly better for IVD and MedSAM performed slightly better for the VB. nnU-Net significantly outperformed other models.

While there are no specific studies that evaluated segmentation of IVD and VB using SAM and MedSAM making a direct comparison difficult, the general trends of increased performance using box rather than point prompts is consistent with studies performed on other types of images. Mazurowski et al. compared point vs. box prompts on 12 different types of medical images (including images from MRI, CT, ultrasound, and X-ray), and found the lowest mean Dice score (~0.5) for the point prompts, and a markedly higher score (~0.6) for the box prompts [46]. In another study on organ segmentation on CT images [47], greater average Dice score was found when using box (~0.8) vs. point prompts (~0.5). We also found that nnU-Net greatly outperformed SAM and MedSAM for both IVD and VB, a finding that agrees with past reports. Although highly variable by modality and task, He et al. [48] reported Dice scores in the order of 0.8 to 0.9 for U-Net, compared to SAM (with point prompts) whose Dice scores had a very wide range from about 0.3 to 0.6. Specifically for IVD and VB, we have previously reported Dice score of ~0.86 [27] using a version of U-Net, and more recent studies have reported value of ~0.95 [49,50]. While the acceptable level of Dice score is subjective, scores above 0.7 has been proposed as the lower limit in brain tumor studies [51,52], but for spine applications, Dice ~0.95 might be considered “good” [53].

Reasons for the better performance of nnU-Net over SAM and MedSAM may include the supervised nature of the nnU-Net, being fine-tuned on the task-specific images and labels, unlike SAM and MedSAM that were trained on a broad set of data and meant to be a generalizable model without a specific task in mind. Although not addressed in this study, another disadvantage of SAM and MedSAM is that these are inherently 2D in nature, and the task of 3D segmentation must be performed as a series of 2D segmentation each providing an input. This not only limits their usefulness and may degrade their relative performance even further when compared to other 3D deep learning models.

Several recent models for general bone segmentation have also been reported. Stojšić et al. [54] reported on a supervised hybrid CNN–transformer model for vertebral body assessment, reporting Dice scores > 0.9 for segmentation. Notable is a model named SegmentAnyBone [55], a variation of SAM that has been fine-tuned for bone segmentation and shown generalizability across different MRI sequences. This model reported Dice scores >0.85, very high for a zero-shot model. Other recent and novel approaches for vertebral body segmentation include deep learning active contours [56,57,58] to avoid fragmentation regions in the outcome.

Our results of similar performance between SAM vs. MedSAM when using box prompts was somewhat unexpected. In the initial development work by Ma et al. [38], MedSAM, with the Dice score reaching 0.9, was reported to be far superior to SAM with a mean Dice score of ~0.7. In an application study involving segmentation of liver cancer on MRI, in the best-case scenario MedSAM (Dice~0.68) outperformed SAM (Dice~0.60) by a small but significant margin [59]. Reasons for such discrepancy may include the lack of the lumbar MRI training data during MedSAM development, although cervical MRI data with vertebral body was included.

**Figure 1:**
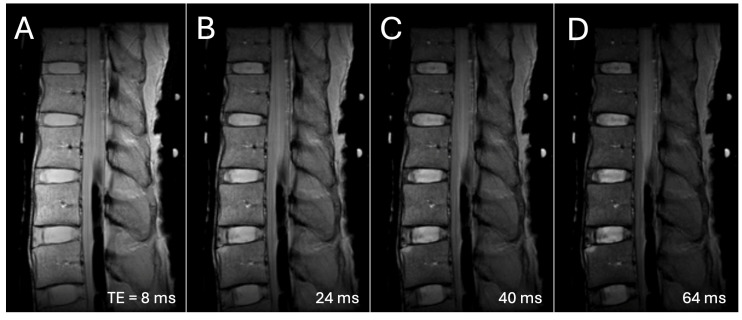
Representative sagittal MR images of a lumbar spine acquired at varying echo times (TE) of (**A**) 8 ms, (**B**) 24 ms, (**C**) 40 ms, and (**D**) 64 ms.

**Figure 2:**
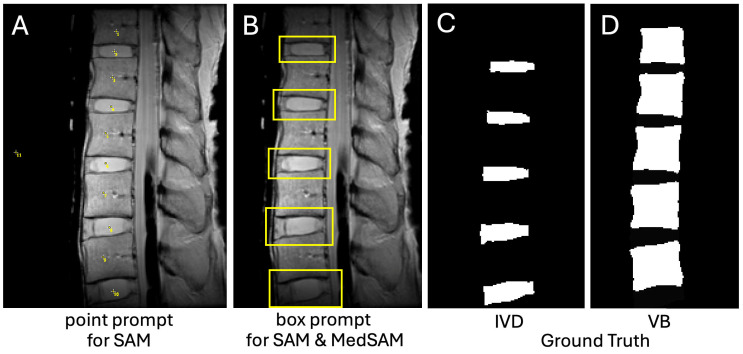
Input prompts needed to perform SAM and MedSAM segmentations. SAM utilized either (**A**) a point prompt where a point or several points can be placed within the region of interest, or (**B**) a bounding box prompt. MedSAM required a bound box prompt. Ground truth images for (**C**) IVD and (**D**) VB segmentation.

**Figure 5:**
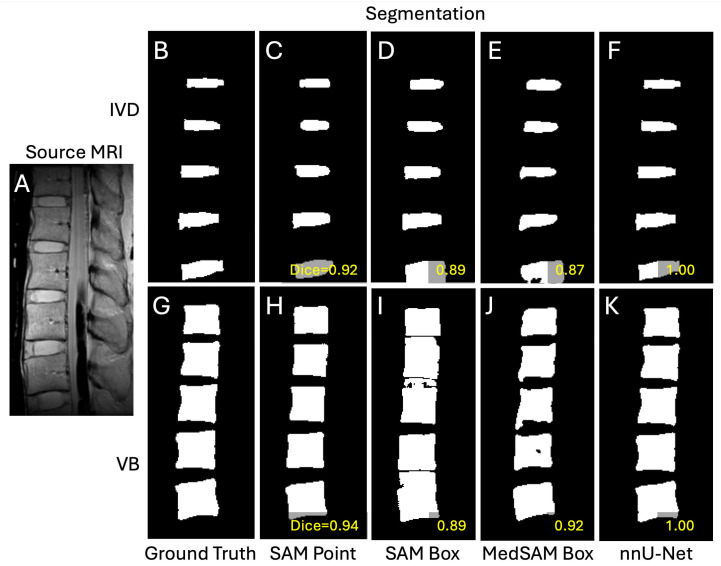
An atypical case where SAM with point prompts had the best outcome. Input image (**A**), ground truths (**B**,**G**), segmentations from DL models of SAM with point prompts (**C**,**H**), SAM with box prompts (**D**,**I**), MedSAM with box prompts (**E**,**J**), and nnU-Net (**F**,**K**). Top row (**B**–**F**) are intervertebral discs and the bottom row (**G**–**K**) are vertebral bodies.

**Figure 6:**
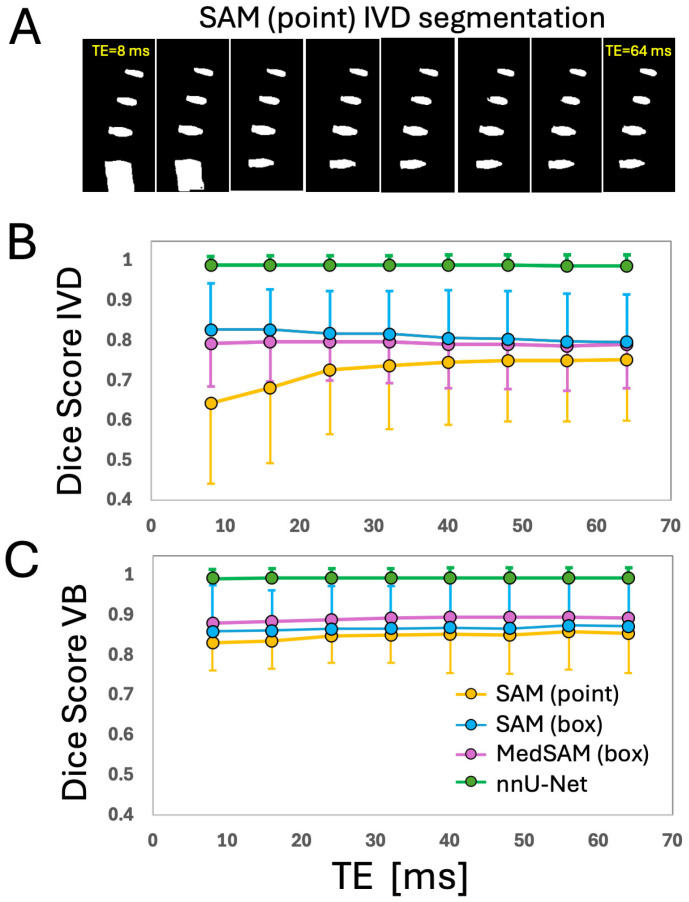
Effect of echo time (TE) on segmentation performance. (**A**) An example of IVD segmentation using SAM with point prompts when source images with different TE were used. Dice scores for (**B**) IVD and (**C**) VB when different DL models and source images TE were used.

While we found that the echo time (TE) of the source image had a significant effect in segmentation performance of all DL models, the differences in Dices scores were generally small. Except for IVD segmentation using SAM with point prompts that showed a marked increase in Dice score at longer TEs (Figure 6A,B), other DL models showed less than 0.03 difference in Dice scores at various TEs. This finding supports the use of these models for MRI images acquired with different scan parameters. The poor performance of SAM with point prompts for IVD at the shortest TE of 8 ms (Dice = 0.64) was surprising at first, since the IVD has the most uniform signal intensity at this TE (Figure 1A) compared to longer TE (Figure 1D) where the periphery of the IVD (annulus pulposus) becomes dark along with the bone marrow within the vertebral bodies. SAM with point prompts tended to over-segment (e.g., Figure 4C,H) when the adjacent region has a similarly high signal intensity (Figure 4A), and this may have contributed to the poor performance.

**Figure 7:**
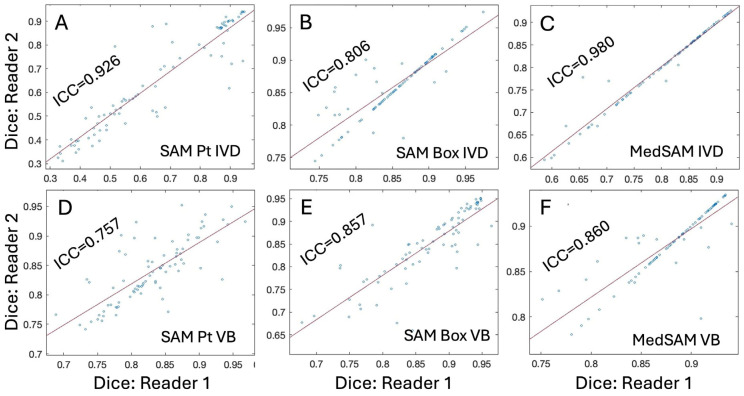
Plots showing inter-reader agreement of Dice scores using intraclass correlation coefficient (ICC) for different tissues and DL models. Correlation plots for IVD are shown on top (**A**–**C**) and the plots for VB are shown on the bottom (**D**–**F**), for the images processed with SAM with point prompts (**A**,**D**), SAM with box prompts (**B**,**E**), and MedSAM with box prompts (**C**,**F**).

We also found generally good-to-excellent inter-reader agreement of SAM and MedSAM models (Figure 7). For SAM with point prompts (Figure 7A,D), despite poor performance, the agreement for IVD was excellent (ICC = 0.926), and for VB (ICC = 0.757) was good. Both SAM with box prompts (Figure 7B,E) and MedSAM (Figure 7C,F) also had good-to-near perfect agreement. Moreover, many data points were along the identity line suggesting that the measurements agreed perfectly.

The integration of robust foundational segmentation models into clinical workflows has the potential to streamline image analysis by automating segmentation of the vertebral bodies and intervertebral discs directly within PACS or radiology workstations. This automation could reduce manual effort (may take tens of minutes per image [60]), improve reproducibility, and enable rapid extraction of quantitative metrics relevant to spinal diseases. In the context of back pain research, the models’ scalability allows for efficient processing of large imaging datasets, which can facilitate the development of imaging biomarkers and supporting population-scale studies that link morphological features to clinical outcomes [61].

This study has several limitations. This study used MRI images of cadaveric specimens instead of in vivo images. The specimens had been dissected and had missing tissues including the skin, paraspinal muscles, etc., that may have resulted in lowered performance of the MedSAM model trained on in vivo images. Images were annotated by a single researcher who is a co-author on the study. It would reduce potential bias if there was an external expert radiologist who also annotated the images. Nonetheless, the segmentation task in this study, while time-consuming, was not a highly difficult one. The number of specimens were small at less than 100, spanning a large range of ages; with a larger training data, the U-Net model performance could be much improved, but this would be irrelevant for the performance of pretrained zero-shot SAM and MedSAM models. Nonetheless, it would be important to have both in vivo images as well as greater numbers for better generalization. This study did not utilize the most advanced or the newest iteration of SAM with improved performance such as modality agnostic SAM [62] and granular box prompt SAM, or assistive techniques to convert point prompts to box prompts [63], which could be considered in the future. For this work, we focused on baseline SAM and MedSAM, which are readily available on a variety of deep learning frameworks but have not been validated for specific anatomies and tasks.

## 5. Conclusions

In conclusion, this study highlights the behavior and performance of SAM and MedSAM zero-shot models for segmentation of IVD and VB in lumbar spine MRI images. Overall, IVD segmentation performed by SAM and MedSAM using box prompts yielded acceptable results, while VB segmentation often had obvious and large errors (that humans would not make) regardless of the prompting methods. The nnU-Net model yielded the most consistent results, on par with past studies. For a carefully chosen and validated targets, the zero-shot models offer key advantages of not needing a task-specific training data and fast adaptation to other anatomies and tasks. The requirement of user input can be a limitation for automated processing but can also provide flexibility when mixed tasks are being performed. Validation of a generalizable segmentation model for lumbar spine MRI would lead to more precise diagnostics, follow-up, enhanced back pain research, and potential cost savings from automated analyses while supporting the broader use of AI and machine learning in healthcare.

## Figures and Tables

**Table 1:** Table of segmentation metrics showing the mean ± standard deviation (SD) of the Dice scores, sensitivity, specificity, and robust Hausdorff distance (RHD) of segmenting intervertebral discs and vertebral bodies using SAM with point prompts, SAM with box prompts, MedSAM with box prompts, and nnU-Net. Red numbers indicate significant differences between DL models.

	Intervertebral Disc							Vertebral Body	
Parameter	Dice	Sensitivity	Specificity	RHD		Dice	Sensitivity	Specificity	RHD
**SAM (Pt), mean ± SD**	0.64 ± 0.20	0.56 ± 0.28	0.995 ± 0.003	7.4 ± 3.6		0.83 ± 0.07	0.77 ± 0.10	0.988 ± 0.014	7.1 ± 1.5
**SAM (box)**	0.83 ± 0.12	0.78 ± 0.14	0.995 ± 0.006	4.1 ± 1.3		0.86 ± 0.05	0.80 ± 0.07	0.989 ± 0.009	6.6 ± 1.3
**MedSAM (box)**	0.79 ± 0.11	0.75 ± 0.14	0.993 ± 0.005	4.6 ± 1.3		0.88 ± 0.04	0.85 ± 0.06	0.986 ± 0.008	5.8 ± 1.0
**nnUNet**	0.99 ± 0.22	0.99 ± 0.03	0.999 ± 0.001	0.5 ± 0.7		0.99 ± 0.01	0.99 ± 0.02	0.999 ± 0.002	0.6 ± 0.9
**Effect of DL model**		**Friedman (Dice, Sensitivity, Specificity) or rmANOVA (RHD) *p*-values**							
**Overall**	**<0.001**	**<0.001**	**<0.001**	**<0.001**		**<0.001**	**<0.001**	**<0.001**	**<0.001**
**Post hoc w/Bonferroni**									
SAM (Pt) vs. SAM (box)	<0.001	<0.001	<0.001	<0.001		<0.001	<0.001	<0.001	<0.001
SAM (Pt) vs. MedSAM	<0.001	<0.001	<0.001	<0.001		<0.001	<0.001	<0.001	<0.001
SAM (Pt) vs. nnU-Net	<0.001	<0.001	<0.001	<0.001		<0.001	<0.001	<0.001	<0.001
SAM (box) vs. MedSAM	<0.001	<0.001	<0.001	<0.001		0.097	<0.05	0.127	<0.05
SAM (box) vs. nnU-Net	<0.001	<0.001	<0.001	<0.001		<0.001	<0.001	<0.001	<0.001
MedSAM vs. nnU-Net	<0.001	0.104	<0.001	<0.001		<0.001	<0.001	<0.001	<0.001

**Table 2:** Table of Friedman *p*-values showing the effect of TE on the Dice scores from each DL model, complementing Figure 5B,C. Red numbers indicate significant differences between DL models.

	IVD Dice	VB Dice
**SAM (Pt)**	<0.001	<0.001
**SAM (box)**	<0.001	<0.001
**MedSAM (box)**	<0.05	<0.001
**nnU-Net**	<0.001	<0.001

## Data Availability

The data that support the findings of this study are not publicly available due to reasons of sensitivity. Anonymized data may be available from the corresponding author upon the review of the request. Data are located in controlled access data storage at the corresponding author’s institution.
